# Supplementary material for: Novel Fluorescence Probe toward Cu2+ Based on Fluorescein Derivatives and Its Bioimaging in Cells
Source: Biosensors (Basel). 2022 Sep 6;12(9):732. doi: 10.3390/bios12090732 (PMC9496130; doi:10.3390/bios12090732)
Supplement: Supplementary file 1 [file biosensors-12-00732-s001.zip › biosensors-1853251-supplementary.pdf]

Novel Fluorescence Probe toward Cu^{2+} Based on Fluorescein Derivatives and Its Bioimaging in Cell

CONTENTS

1. Synthesis of Probe A5 S-2

2. MTT Assay S-5

3. Visual Detection S-5

1. Synthesis of Probe A5

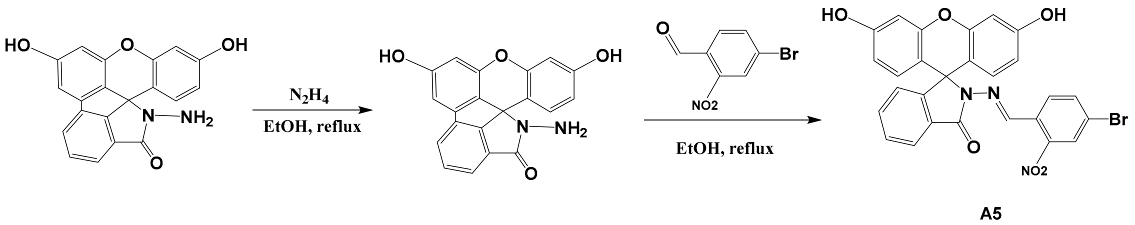

Scheme S1 Synthesis route of probe **A5**

Fluorescein hydrazine was synthesized from fluorescein and hydrazine according to the literature. Fluorescein hydrazine (3.39 g, 9.78 mmol) and 4-Bromo-2-nitrobenzaldehyde, (1.50 g, 6.52 mmol) were dissolved in 50 mL of ethanol, refluxed for 3 h, cooled to room temperature after the reaction. The precipitate was filtered out, washed several times with absolute ethanol, and the pale-yellow solid was obtained and placed in a dark place at 5 °C for use.

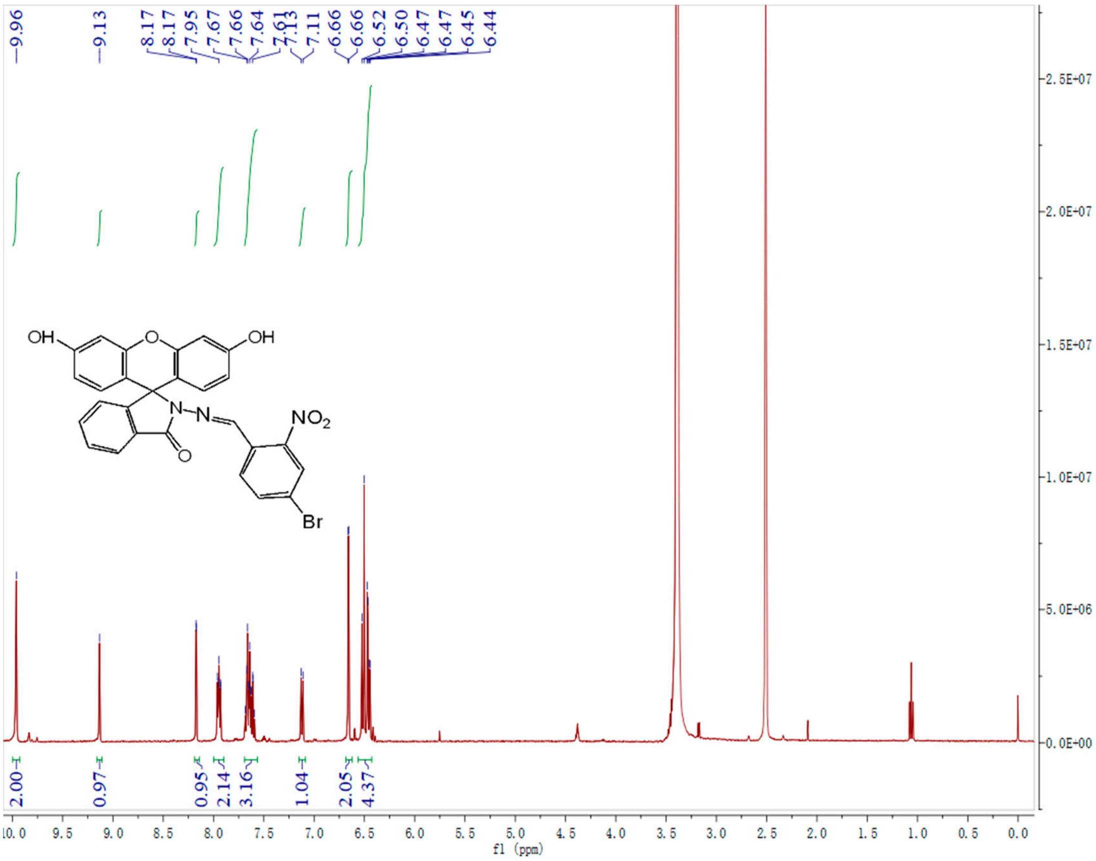

Figure S1 ^{1}H NMR spectrum of compound A5 in d_6-DMSO.

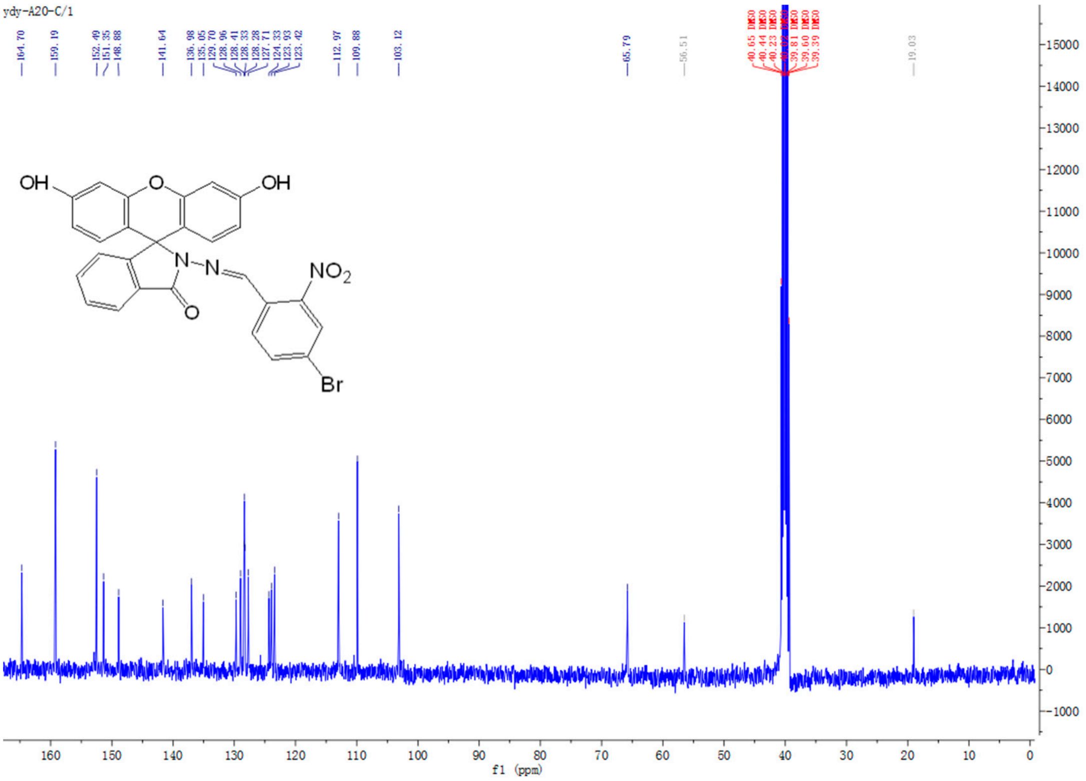

Figure S2 ^{13}C NMR spectrum of compound A5 in d_6-DMSO.

Mass Spectrum SmartFormula Report

Analysis Info

Analysis Name	C:\Users\ÀÌÐËÐË\Desktop\ÐÂ½¨ÎÄ¼þ¼Ð\ÁõÁÖ\ÁõÁÖ\Ó«¹âÈØêÂÓëÈ©ÀàµÄ·Ó¦\Ó«¹âÈØêÂÓë4-äà-2-ïõ»ù±½¼×È©·Ó¦	Acquisition Date	2018/4/27 15:51:19
Method	tune_low_2000.m / C:A-20\\ybz\tengxin_A-20.d	Operator	service
Sample Name	ld016-003	Instrument / Ser#	micrOTOF-Q II 10280
Comment			

Acquisition Parameter

Source Type	ESI	Ion Polarity	Positive	Set Nebulizer	0.4 Bar
Focus	Not active	Set Capillary	4500 V	Set Dry Heater	180 °C
Scan Begin	50 m/z	Set End Plate Offset	-500 V	Set Dry Gas	4.0 l/min
Scan End	3000 m/z	Set Collision Cell RF	110.0 Vpp	Set Divert Valve	Source

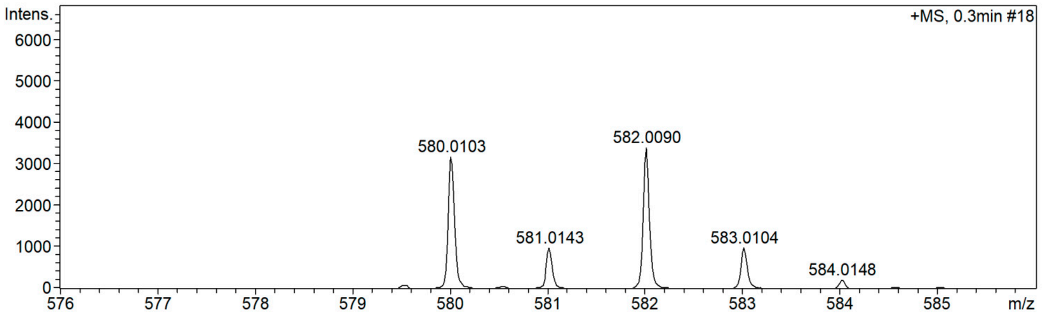

Meas. m/z	#	Formula	Score	m/z	err [mDa]	err [ppm]	mSigma	rdb	e¡¥ Conf	N-Rule
580.0103	1	C 27 H 16 Br N 3 Na O 6	100.00	580.0115	1.1	2.0	14.5	20.5	even	ok

Figure S3 ESI-MS spectrum of A5

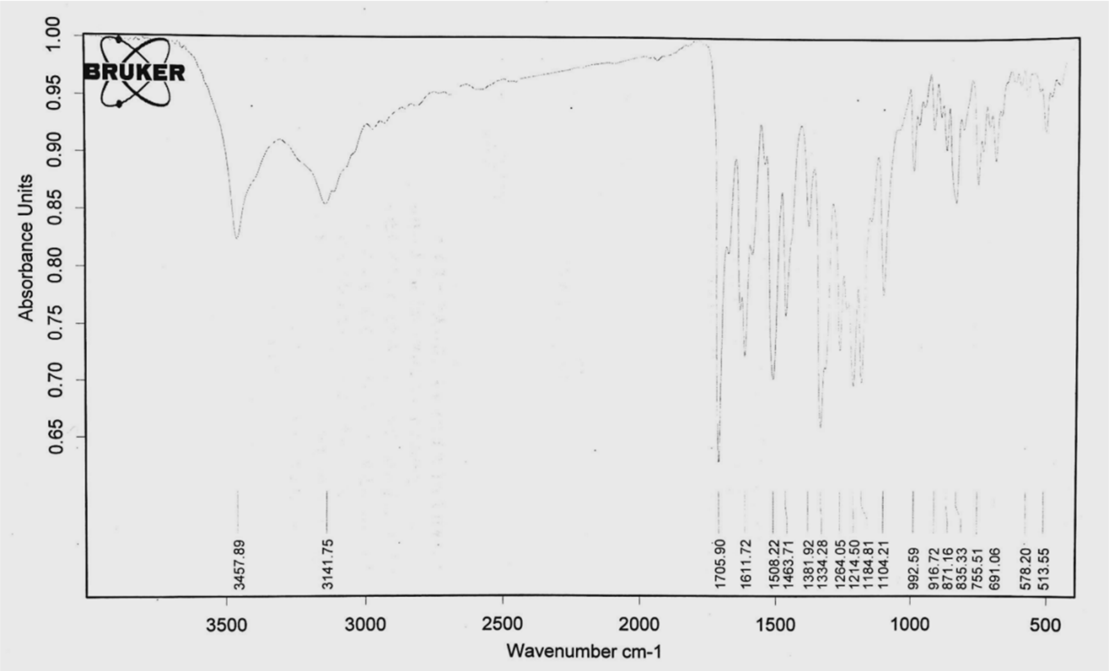

Figure S4 IR spectrum of A5

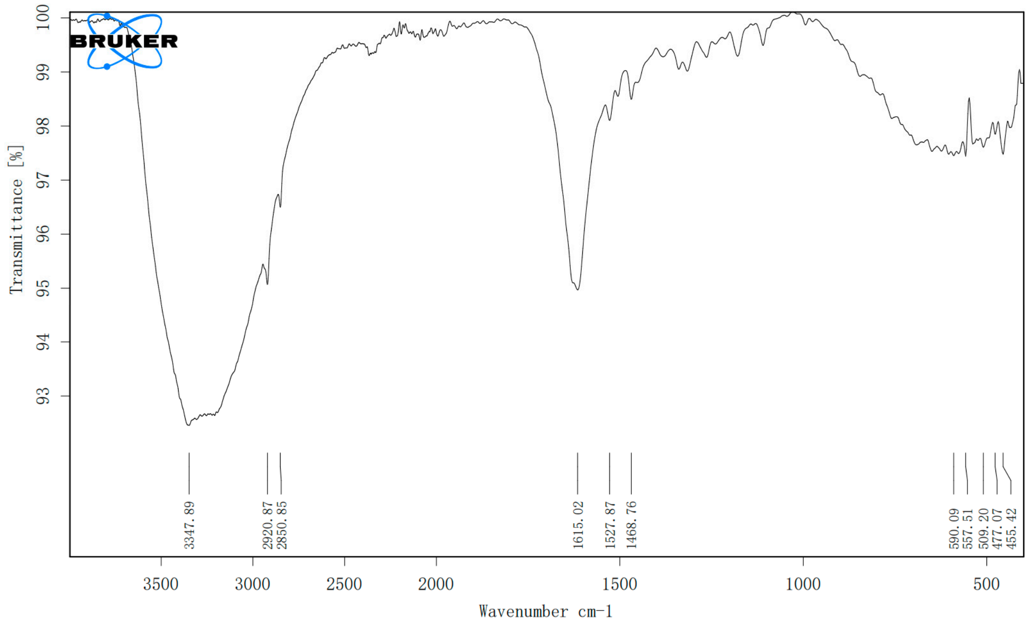

Figure S5 IR spectrum of A5 and Cu^{2+}

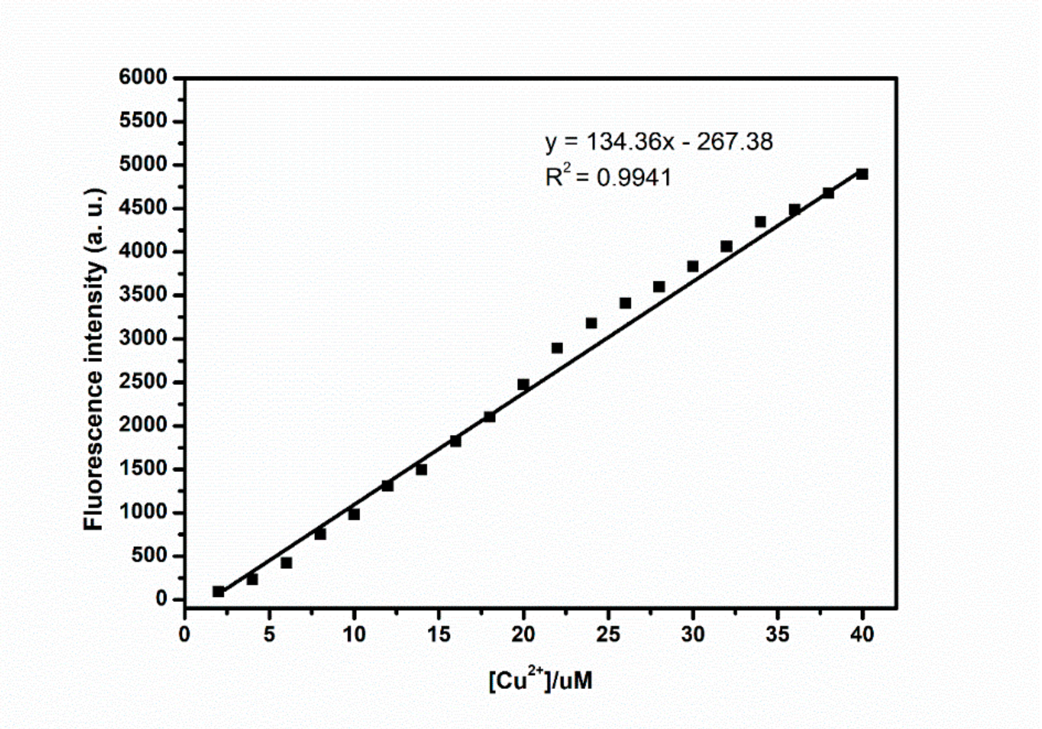

Figure S6 The linear correlation between the maximum fluorescence intensity (525 nm) and the concentration of Cu^{2+}; λex = 440 nm.

3. MTT Assay

3-(4,5-Dimethylthiazol-2-yl)-2,5-diphenyltetrazolium bromide (MTT) assay. MCF-7 cells were seeded in 96-well plates at a density of 5000 cells/well. Plates were maintained at 37 °C in a 5% CO_2/ 95% air incubator for 24 hours. Then, the culture medium in each well was replaced by fresh medium containing different concentrations of probe **A5**. After 24 hours treatment, into each well, 15μL MTT solutions (5 mg/mL in phosphate buffer solution) were added. After 4 hours incubation at 37 °C, the absorbance of each wells at 490 nm was recorded by the Elx800 Absorbance Microplate Reader.

Table S1. The cell viability of different concentration of probe **A5**

**Sample**	2.5 μM/L	5 μM/L	10 μM/L	20 μM/L	40 μM/L
**A5**	98.7	91.0	90.7	82.0	73.3

4. Visual Detection

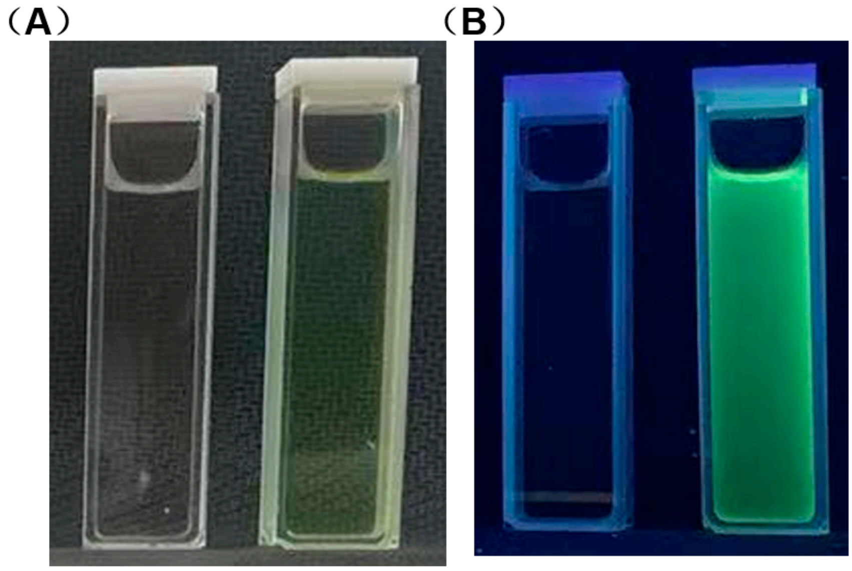

Figure S7 (A) Pictures under ambient light. (B) Pictures under 365 UV lamp.

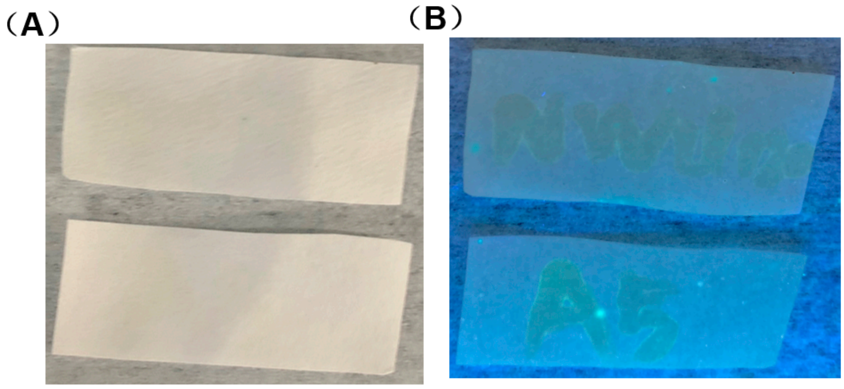

Figure S8 Photographs of A5 and after Cu^{2+} was written on a test paper, (A) Pictures under ambient light. (B) Pictures under 365 UV lamp.
